# Cured Symptoms

**Published:** 1896-11

**Authors:** C. M. Boger

**Affiliations:** Parkersburg, W. Va.


### CURED SYMPTOMS.

C. M. BOGER, M. D., PARKERSBURG, W. VA.

1. Balanorrhea, profuse discharge of pus, with smarting and burning. ℞ Jacaranda $^{3x}$. Cured.

2. Sensation as if the sternum were swelled, with painful soreness. ℞ Osmium $^{9m}$. Cured.

3. Cutting in the spleen (Bell., Crotal., Dulc., Verb.), from having stood in a draft, phlegmatic temperament, abuse of alcohol. ℞ Bell.$^{3x}$. Cured.

4. Sensation of drawing together of ears internally; better from boring finger into ears (Bov., Colo., Lachn., Mez.). ℞ Mez.$^{50m}$. Cured.

5. Sensation as if struck in the eyes with the hairs of a brush, with a boring below left scapula, during Bright's disease. ℞ Agar-an.$^{2m}$. Removed symptoms.

6. Sensation of burning heat and scratching in the eyes, *only when lying down* (Carb-v., Znc.); prevents rest at night, conjunctiva very dry, almost no secretion; this remedy restored secretion, which became at first purulent, then normal. ℞ Carb-v.$^{50m}$. Cured.

7. Leaden heaviness in stomach, worse after eating, especially sour things; *quivering* in the *left hypochondrium* and *left ovary;* vertigo; frightful dreams. ℞ Arg-nit.$^{40m}$. Cured.

